# Supplementary material for: Gradual compaction of the nascent peptide during cotranslational folding on the ribosome
Source: eLife. 2020 Oct 27;9:e60895. doi: 10.7554/eLife.60895 (PMC7593090; doi:10.7554/eLife.60895)
Supplement: Supplementary file 1. — Table 1. ACF (each average of N ≥ 8) fits of HemK constructs in solution. Table 2 results of empirical fits of PET-FCS ACF (each ACF an average of N ≥ 8) for RNCs. Table 3 results of global fitting of the free Trp titration (dataset A) to model 5e-H. Table 4 results of global fitting of the free Trp titration (dataset A) to model 5e-O. Table 5. Results of global fitting of the dataset B to the model 5e-H. Table 6. Results of global fitting of the dataset B to the model 5e-O. Table 7. Upper and lower boundaries of each rate parameter of model 5e-H and 5e-O global fitting. Table 8. Free-energy calculations for all RNC constructs using rates derived from the 5e-H model. Table 9. Free-energy calculations for all RNC constructs from the 5e-O model rates. Table 10. PET-FCS constructs aa sequences N- to C-terminus. Table 11. Force-profile construct of wt HemK full-length, aa sequence N- to C-terminus. [file elife-60895-supp1.docx]

**Supplementary Tables**

Gradual Compaction of the Nascent Peptide During Cotranslational Folding on the Ribosome

Marija Liutkute, Manisankar Maiti, Ekaterina Samatova, Jörg Enderlein and Marina V. Rodnina

Table 1 ACF (each average of N≥8) fits of HemK constructs in solution. All fit errors are calculated as standard error of the mean and are <10 %. Rates k_1_ and k_d_ are in s^-1^. τ_1_ and τ_d1_ are relaxation time constants of the respective exponents, in s, τ=1/k. N is the average number of molecules in the confocal volume and c_1_ is the amplitude of the fast relaxation time.

| HemK | c_1_ | τ_1_ x10^-7^ | k_1_ x10^6^ | N | τ_d1_ x10^-5^ | k_d_ x10^3^ |
| --- | --- | --- | --- | --- | --- | --- |
| 0% glycerol |  |  |  |  |  |  |
| 70 W6 |  |  |  | 0.83 | 5.16 | 19.38 |
| 70 W6F |  |  |  | 0.83 | 5.42 | 18.43 |
| 70 4xA W6 |  |  |  | 0.85 | 5.78 | 17.30 |
| 70 4xA W6F |  |  |  | 0.83 | 5.59 | 17.89 |
| 14 W6 |  |  |  | 0.84 | 5.88 | 17.00 |
| 14 W6F |  |  |  | 0.86 | 6.61 | 15.13 |
| 50% glycerol |  |  |  |  |  |  |
| 70 W6 |  |  |  | 0.94 | 30.78 | 3.25 |
| 70 W6F |  |  |  | 0.95 | 33.87 | 2.95 |
| 70 4xA W6 |  |  |  | 0.95 | 30.40 | 3.29 |
| 70 4xA W6F |  |  |  | 0.95 | 29.10 | 3.44 |
| 14 W6 | 0.15 | 5.13 | 1.95 | 0.97 | 45.09 | 2.22 |
| 14 W6F |  |  |  | 0.97 | 60.31 | 1.66 |

Table 2 Results of empirical fits of PET-FCS ACF (each ACF an average of N≥8) for RNCs. All fit errors are calculated as standard error of the mean and are indicated in the table. Rates (k_x_) are in s^-1^. τ_1_, τ_2_, τ_f_ and τ_d_ are relaxation time constants of the respective exponents, in s, τ=1/k.

| Construct | c_1_ | k_1_  x10^6^ | τ_1_ x10^-7^ | c_2_ | k_2_ x10^5^ | τ_2_ x10^-6^ | F | k_f_ x10^4^ | τ_f_  x10^-5^ | N | k_d_ x10^3^ | τ_d_ |
| --- | --- | --- | --- | --- | --- | --- | --- | --- | --- | --- | --- | --- |
| 70 W6 | 0.17 ± 0.01 | 2.58  ± 0.21 | 3.87 | 0.68 ± 0.01 | 4.55 ± 0.09 | 2.20 | 0.18 ± 0.003 | 2.29 ± 0.09 | 4.38 | 0.93 ± 0.003 | 1.05 ± 0.008 | 0.001 |
| 70 W6F | 0.14 ± 0.01 | 2.29 ± 0.19 | 4.36 | 0.62 ± 0.01 | 4.59 ± 0.09 | 2.18 | 0.13 ± 0.002 | 2.45 ± 0.10 | 4.09 | 0.93 ± 0.002 | 0.95 ± 0.005 | 0.001 |
| 70 4xA W6 | 0.18 ± 0.01 | 2.67 ± 0.16 | 3.74 | 0.69 ± 0.01 | 4.51 ± 0.07 | 2.22 | 0.16 ± 0.002 | 2.45 ± 0.09 | 4.08 | 0.93 ± 0.002 | 0.99 ± 0.006 | 0.001 |
| 70 4xA W6F | 0.13 ± 0.01 | 2.61  ± 0.23 | 3.84 | 0.61 ± 0.01 | 4.68 ± 0.09 | 2.13 | 0.12 ± 0.002 | 2.86 ± 0.14 | 3.49 | 0.92 ± 0.002 | 0.93 ± 0.005 | 0.001 |
| 102 W6 | 0.20 ± 0.01 | 3.30  ± 0.19 | 3.03 | 0.61 ± 0.01 | 4.29 ± 0.07 | 2.33 | 0.18 ± 0.002 | 2.36 ± 0.09 | 4.23 | 0.94 ± 0.002 | 0.95 ± 0.007 | 0.001 |
| 102 W6F | 0.14 ± 0.01 | 2.91 ± 0.23 | 3.43 | 0.56 ± 0.01 | 4.66 ± 0.09 | 2.15 | 0.13 ± 0.002 | 2.54 ± 0.11 | 3.94 | 0.93 ± 0.002 | 0.93 ± 0.005 | 0.001 |
| 102 loop | 0.13 ± 0.01 | 2.89 ± 0.24 | 3.46 | 0.57 ± 0.01 | 4.33 ± 0.08 | 2.31 | 0.14 ± 0.002 | 2.54 ± 0.12 | 3.93 | 0.93 ± 0.002 | 0.86 ± 0.005 | 0.001 |
| 112 loop | 0.14 ± 0.01 | 2.72 ± 0.21 | 3.68 | 0.54 ± 0.01 | 4.24 ± 0.08 | 2.36 | 0.13 ± 0.002 | 2.47 ± 0.11 | 4.05 | 0.93 ± 0.002 | 0.88 ± 0.005 | 0.001 |
| 112 W6 | 0.19 ± 0.01 | 3.61 ± 0.19 | 2.77 | 0.48 ± 0.01 | 4.41 ± 0.08 | 2.27 | 0.10 ± 0.002 | 2.79 ± 0.16 | 3.59 | 0.93 ± 0.002 | 0.84 ± 0.004 | 0.001 |
| 112 W6F | 0.14 ± 0.01 | 2.94 ± 0.22 | 3.40 | 0.52 ± 0.01 | 4.45 ± 0.09 | 2.25 | 0.11 ± 0.002 | 2.51 ± 0.13 | 3.98 | 0.93 ± 0.002 | 0.91 ± 0.005 | 0.001 |
| 112 4xA W6 | 0.20 ± 0.01 | 2.16 ± 0.13 | 4.63 | 0.59 ± 0.01 | 3.73 ± 0.08 | 2.68 | 0.21 ± 0.003 | 1.76 ± 0.06 | 5.68 | 0.95 ± 0.003 | 1.00 ± 0.008 | 0.001 |
| 112 4xA W6F | 0.12 ± 0.01 | 2.52 ± 0.23 | 3.97 | 0.43 ± 0.01 | 3.94 ± 0.97 | 2.54 | 0.11 ± 0.002 | 2.30 ± 0.13 | 4.35 | 0.93 ± 0.002 | 0.86 ± 0.005 | 0.001 |

Table 3 Results of global fitting of the free Trp titration (dataset A) to model 5e-H. Rates that were linked during global fit are shown in the same cell shades. All reported rates (k) are in μs⁻¹, except the Wd and Wc k on rates that are in mM⁻¹ μs⁻¹, rates where SEM exceeds value are given as not significant (n.s.). A covariance matrix derived using nonlinear regression algorithms is used to estimate the standard errors (SEM) by the Kintek Explorer software.

| Construct | k | D ↔ Rd | C ↔ Rc | D ↔ Wd | C ↔ Wc | D ↔ C |
| --- | --- | --- | --- | --- | --- | --- |
| 70 W6F | on | 139 ± 14 | n.s. | 0.010 ± 0.004 | ~ 0 | n.s. |
|  | off | n.s. | n.s. | 2.2 ± 0.2 | 2.2 ± 0.2 | 0.50 ± 0.07 |
| 102 W6F | on | 461 ± 69 | n.s. | 0.03 ± 0.01 | ~ 0 | n.s. |
|  | off | n.s. | 0.51 ± 0.09 | 2.2 ± 0.2 | 2.2 ± 0.2 | 1.9 ± 0.2 |

Table 4 Results of global fitting of the free Trp titration (dataset A) to model 5e-O. Legend as in Supplementary Table 3.

| Construct | k | D ↔ R | C ↔ R | D ↔ W | C ↔ W | D ↔ C |
| --- | --- | --- | --- | --- | --- | --- |
| 70 W6F | on | 83 ± 17 | 0.5 ± 0.06 | 0.006 ± 0.003 | ~ 0 | n.s. |
|  | off | n.s. | n.s. | 2 ± 0.1 | 2 ± 0.1 | n.s. |
| 102 W6F | on | 85 ± 15 | 0.5 ± 0.05 | 0.007 ± 0.003 | ~ 0 | n.s. |
|  | off | n.s. | n.s. | 2 ± 0.1 | 2 ± 0.1 | n.s. |

Table 5 Results of global fitting of the dataset B to the model 5e-H. Rates (k) are reported in μs⁻¹, rates linked during global fit are shown in the same cell shade; locked values are in red. A covariance matrix derived using nonlinear regression algorithms is used to estimate the standard errors (SEM) by the Kintek Explorer software.

| Construct | k | D ↔ Rd | C ↔ Rc | D ↔ Wd | C ↔ Wd | D ↔ C |
| --- | --- | --- | --- | --- | --- | --- |
| 70 wt | on | 9.7 ± 1.3 | 0.24 ± 0.02 | 7.1 ± 1.2 | 0.04 ± 0.02 | 1.4 ± 0.1 |
|  | off | 0.30 ± 0.02 | 0.30 ± 0.02 | 2.2 | 2.2 | 0.18 ± 0.01 |
| 70 4xA | on | 9.7 ± 1.3 | 0.24 ± 0.02 | 7.1 ± 1.2 | 0.04 ± 0.02 | 1.4 ± 0.1 |
|  | off | 0.30 ± 0.02 | 0.30 ± 0.02 | 2.2 | 2.2 | 0.18 ± 0.01 |
| 102 wt | on | 4.7 ± 0.2 | 0.24 ± 0.02 | 3.4 ± 0.2 | 0.04 ± 0.02 | 0.50 ± 0.04 |
|  | off | 0.30 ± 0.02 | 0.30 ± 0.02 | 2.2 | 2.2 | 0.14 ± 0.01 |
| 102 loop | on | 4.7 ± 0.2 | 0.24 ± 0.02 |  |  | 5.04 ± 0.73 |
|  | off | 0.30 ± 0.02 | 0.30 ± 0.02 |  |  | 1.4 ± 0.2 |
| 112 loop | on | 11.5 ± 0.5 | 0.24 ± 0.02 |  |  | 3.7 ± 0.4 |
|  | off | 0.30 ± 0.02 | 0.30 ± 0.02 |  |  | 0.41 ± 0.03 |
| 112 wt | on | 11.5 ± 0.5 | 0.24 ± 0.02 | 8.4 ± 0.4 | 0.04 ± 0.02 | 0.04 ± 0.11 |
|  | off | 0.30 ± 0.02 | 0.30 ± 0.02 | 2.2 | 2.2 | 0.005 ± 0.018 |
| 112 4xA | on | 11 ± 2.9 | 0.22 ± 0.08 | 8.1 ± 2.5 | 0.04 ± 0.07 | 2.2 ± 0.1 |
|  | off | 0.30 ± 0.02 | 0.30 ± 0.02 | 2.2 | 2.2 | 0.23 ± 0.05 |

Table 6 Results of global fitting of the dataset B to the model 5e-O. The legend is the same as in Supplementary Table 5.

| Construct | k | D ↔ R | C ↔ R | D ↔ W | C ↔ W | D ↔ C |
| --- | --- | --- | --- | --- | --- | --- |
| 70 wt | on | 22 ± 3.3 | 0.37 ± 0.02 | 4.2 ± 3 | 0.07 ± 0.03 | 0.975 ± 0.3 |
|  | off | 0.3 ± 0.02 | 0.3 ± 0.02 | 2 | 2 | 0.0167 ± 0.02 |
| 70 4xA | on | 22 ± 3.3 | 0.37 ± 0.02 | 4.2 ± 3 | 0.07 ± 0.03 | 0.975 ± 0.3 |
|  | off | 0.3 ± 0.02 | 0.3 ± 0.02 | 2 | 2 | 0.0167 ± 0.02 |
| 102 wt | on | 25 ± 1.2 | 0.37 ± 0.02 | 4.8 ± 1 | 0.07 ± 0.03 | 0.13 ± 0.3 |
|  | off | 0.3 ± 0.02 | 0.3 ± 0.02 | 2 | 2 | 0.002 ± 0.02 |
| 102 loop | on | 25 ± 1.2 | 0.37 ± 0.02 |  |  | 4.39 ± 0.5 |
|  | off | 0.3 ± 0.02 | 0.3 ± 0.02 |  |  | 0.0653 ± 0.03 |
| 112 loop | on | 29 ± 1.3 | 0.37 ± 0.02 |  |  | 3.44 ± 0.5 |
|  | off | 0.3 ± 0.02 | 0.3 ± 0.02 |  |  | 0.0442 ± 0.03 |
| 112 wt | on | 29 ± 1.3 | 0.37 ± 0.02 | 5.6 ± 1 | 0.07 ± 0.03 | 0.0002 ± 0.3 |
|  | off | 0.3 ± 0.02 | 0.3 ± 0.02 | 2 | 2 | 0.000002 ± 0.02 |
| 112 4xA | on | 24 ± 8.2 | 0.35 ± 0.02 | 4.7 ± 8 | 0.07 ± 0.01 | 1.62 ± 0.4 |
|  | off | 0.3 ± 0.02 | 0.3 ± 0.02 | 2 | 2 | 0.0232 ± 0.1 |

Table 7 Upper and lower boundaries of each rate parameter computed with a χ^2^/_min_χ^2^ threshold: 0.8333. Rates linked during global fit are shown in the same cell shade; locked values are in red.

| Construct | Model 5e-O | | | **Elemental Rate** | Model 5e-H | | |
| --- | --- | --- | --- | --- | --- | --- | --- |
|  | Best-fit Value | Lower Boundary | Upper Boundary |  | Best-fit Value | Lower Boundary | Upper Boundary |
| 70wt | 21.8 | 17.4 | 28.9 | k_on_ D ↔ R_(d)_ | 9.7 | 6.6 | 16.3 |
|  | 0.37 | 0.35 | 0.39 | k_on_ C ↔ R_(c)_ | 0.24 | 0.15 | 0.30 |
|  | 0.31 | 0.25 | 0.46 | k_off_ D/C ↔ R_(d/c)_ | 0.30 | 0.17 | 0.45 |
|  | 4.2 | 2.6 | 6.3 | k_on_ D ↔ W_(d)_ | 7.1 | 5.4 | 9.4 |
|  | 0.07 | 0.04 | 0.10 | k_on_ C ↔ W_(c)_ | 0.04 | 0.001 | 0.13 |
|  | 2 | n/a | n/a | k_off_ D/C ↔ W_(d/c)_ | 2.2 | n/a | n/a |
|  | 0.98 | 0.45 | 2.83 | k_on_ D ↔ C | 1.4 | 0.56 | 2.8 |
|  | 0.02 | 0.01 | 0.04 | k_off_ D ↔ C | 0.18 | 0.07 | 0.35 |
|  |  |  |  |  |  |  |  |
| 70 4xA | 21.8 | 17.4 | 28.9 | k_on_ D ↔ R_(d)_ | 9.7 | 6.6 | 16.3 |
|  | 0.37 | 0.35 | 0.39 | k_on_ C ↔ R_(c)_ | 0.24 | 0.15 | 0.30 |
|  | 0.31 | 0.25 | 0.46 | k_off_ D/C ↔ R_(d/c)_ | 0.30 | 0.17 | 0.45 |
|  | 4.2 | 2.6 | 6.3 | k_on_ D ↔ W_(d)_ | 7.1 | 5.4 | 9.4 |
|  | 0.07 | 0.04 | 0.10 | k_on_ C ↔ W_(c)_ | 0.04 | 0.001 | 0.13 |
|  | 2 | n/a | n/a | k_off_ D/C ↔ W_(d/c)_ | 2.2 | n/a | n/a |
|  | 0.98 | 0.45 | 2.83 | k_on_ D ↔ C | 1.4 | 0.56 | 2.8 |
|  | 0.02 | 0.01 | 0.04 | k_off_ D ↔ C | 0.18 | 0.07 | 0.35 |
|  |  |  |  |  |  |  |  |
| 102 wt | 25 | 20 | 35.2 | k_on_ D ↔ R_(d)_ | 4.7 | 3.0 | 6.7 |
|  | 0.37 | 0.35 | 0.39 | k_on_ C ↔ R_(c)_ | 0.24 | 0.15 | 0.30 |
|  | 0.31 | 0.25 | 0.46 | k_off_ D/C ↔ R_(d/c)_ | 0.30 | 0.17 | 0.45 |
|  | 4.9 | 2.97 | 7.2 | k_on_ D ↔ W_(d)_ | 3.4 | 2.7 | 4.1 |
|  | 0.07 | 0.04 | 0.10 | k_on_ C ↔ W_(c)_ | 0.04 | 0.001 | 0.13 |
|  | 2 | n/a | n/a | k_off_ D/C ↔ W_(d/c)_ | 2.2 | n/a | n/a |
|  | 0.13 | 8x10^-7^ | 1.8 | k_on_ D ↔ C | 0.50 | 0.08 | 0.88 |
|  | 0.002 | 1x10^-8^ | 0.025 | k_off_ D ↔ C | 0.14 | 0.03 | 0.24 |
|  |  |  |  |  |  |  |  |
| 102 loop | 25 | 20 | 35.2 | k_on_ D ↔ R_(d)_ | 4.7 | 3.0 | 6.7 |
|  | 0.37 | 0.35 | 0.39 | k_on_ C ↔ R_(c)_ | 0.24 | 0.15 | 0.30 |
|  | 0.31 | 0.25 | 0.46 | k_off_ D/C ↔ R_(d/c)_ | 0.30 | 0.17 | 0.45 |
|  | 4.4 | 3.5 | 7.7 | k_on_ (D ↔ C) | 5.04 | 1.8 | 69.8 |
|  | 0.065 | 0.056 | 0.089 | k_off_ (D ↔ C) | 1.4 | 0.54 | 21.1 |
|  |  |  |  |  |  |  |  |
| 112 loop | 29 | 23.2 | 40.7 | k_on_ D ↔ R_(d)_ | 11.5 | 7.8 | 20.2 |
|  | 0.37 | 0.35 | 0.39 | k_on_ C ↔ R_(c)_ | 0.24 | 0.15 | 0.30 |
|  | 0.31 | 0.25 | 0.46 | k_off_ D/C ↔ R_(d/c)_ | 0.30 | 0.17 | 0.45 |
|  | 3.4 | 2.8 | 6.0 | k_on_ D ↔ C | 3.7 | 0.91 | 8.1 |
|  | 0.044 | 0.038 | 0.066 | k_off_ D ↔ C | 0.41 | 0.11 | 0.71 |
|  |  |  |  |  |  |  |  |
| 112 wt | 29 | 23.2 | 40.7 | k_on_ D ↔ R_(d)_ | 11.5 | 7.8 | 20.2 |
|  | 0.37 | 0.35 | 0.39 | k_on_ C ↔ R_(c)_ | 0.24 | 0.15 | 0.30 |
|  | 0.31 | 0.25 | 0.46 | k_off_ D/C ↔ R_(d/c)_ | 0.30 | 0.17 | 0.45 |
|  | 5.6 | 3.4 | 8.8 | k_on_ D ↔ W_(d)_ | 8.4 | 6.35 | 11.8 |
|  | 0.07 | 0.04 | 0.10 | k_on_ C ↔ W_(c)_ | 0.04 | 0.001 | 0.13 |
|  | 2 | n/a | n/a | k_off_ D/C ↔ W_(d/c)_ | 2.2 | n/a | n/a |
|  | 0.0002 | 2x10^-8^ | 1.6 | k_on_ D ↔ C | 0.04 | 5x10^-5^ | 0.66 |
|  | 2x10^-6^ | 2x10^-10^ | 0.02 | k_off_ D ↔ C | 0.005 | 6x10^-6^ | 0.06 |
|  |  |  |  |  |  |  |  |
| 112 4xA | 24.1 | 17.4 | 40 | k_on_ D ↔ R_(d)_ | 11 | 7.5 | 19.4 |
|  | 0.35 | 0.33 | 0.36 | k_on_ C ↔ R_(c)_ | 0.22 | 0.14 | 0.28 |
|  | 0.31 | 0.25 | 0.46 | k_off_ D/C ↔ R_(d/c)_ | 0.30 | 0.17 | 0.45 |
|  | 4.7 | 2.9 | 8.3 | k_on_ D ↔ W_(d)_ | 8.1 | 6.1 | 10.1 |
|  | 0.067 | 0.041 | 0.095 | k_on_ C ↔ W_(c)_ | 0.04 | 0.001 | 0.12 |
|  | 2 | n/a | n/a | k_off_ D/C ↔ W_(d/c)_ | 2.2 | n/a | n/a |
|  | 1.6 | 0.66 | 4.7 | k_on_ D ↔ C | 2.2 | 1.6 | 3.4 |
|  | 0.023 | 0.012 | 0.045 | k_off_ D ↔ C | 0.2 | 0.18 | 0.34 |

Table 8 Free energy calculations for all RNC constructs using rates derived from the 5e-H model. Elemental rate SEM from the kinetic fits are propagated through equation 2 (Methods).

| Construct | ΔG°_D_–ΔG^‡^, kJ mol^-1^ | ΔG°_C_–ΔG^‡^, kJ mol^-1^ | ΔG°_D_–ΔG°_C_, kJ mol^-1^ |
| --- | --- | --- | --- |
| 70 wt | 37.5 ± 0.0001 | 42.5 ± 0.0001 | 5.0 ± 0.0001 |
| 70 4xA | 37.5 ± 0.0001 | 42.5 ± 0.0001 | 5.0 ± 0.0001 |
| 102 wt | 40.0 ± 0.0001 | 43.2 ± 0.0001 | 3.1 ± 0.0001 |
| 102 loop | 34.4 ± 0.0001 | 37.5 ± 0.0001 | 3.1 ± 0.0001 |
| 112 loop | 35.1 ± 0.0001 | 40.5 ± 0.0001 | 5.4 ± 0.0001 |
| 112 wt | 46.2 ± 0.003 | 51.3 ± 0.004 | 5.1 ± 0.005 |
| 112 4xA | 36.4 ± 0.00005 | 42.0 ± 0.0002 | 5.5 ± 0.0002 |

Table 9 Free energy calculations for all RNC constructs from the 5e-O model rates. Elemental rate SEM from the kinetic fits are propagated through equation 2 (Methods).

| Construct | ΔG°_D_–ΔG^‡^, kJ mol^-1^ | ΔG°_C_–ΔG^‡^, kJ mol^-1^ | ΔG°_D_–ΔG°_C_, kJ mol^-1^ |
| --- | --- | --- | --- |
| 70 wt | 38.4 ± 0.0003 | 48.4 ± 0.001 | 10.0 ± 0.001 |
| 70 4xA | 38.4 ± 0.0003 | 48.4 ± 0.0000 | 10.0 ± 0.0003 |
| 102 wt | 43.3 ± 0.002 | 53.6 ± 0.01 | 10.3 ± 0.01 |
| 102 loop | 34.7 ± 0.0001 | 45.0 ± 0.0005 | 10.3 ± 0.0005 |
| 112 loop | 35.3 ± 0.0001 | 46.0 ± 0.0007 | 10.7 ± 0.0007 |
| 112 wt | 59.2 ± 1.5 | 70.5 ± 10 | 11.3 ± 10.1 |
| 112 4xA | 37.2 ± 0.0002 | 47.6 ± 0.004 | 10.4 ± 0.004 |

Table 10 PET-FCS constructs aa sequences N- to C-terminus.

| **HemK** | **N- to C-terminus 112 aa constructs** |
| --- | --- |
| **wt** | MEFQHWLREA ISQLQASESP RRDAEILLEH VTGKGRTFIL AFGETQLTDE QCQQLDALLT RRRDGEPIAH LTGVREFFSL PLFVSPATLI PRPDTECLVE QALARLPEQP CR |
| **wt W6F** | MEFQHFLREA ISQLQASESP RRDAEILLEH VTGKGRTFIL AFGETQLTDE QCQQLDALLT RRRDGEPIAH LTGVREFFSL PLFVSPATLI PRPDTECLVE QALARLPEQP CR |
| **looped** | MEFQHFLREA ISQLQASESP RRDAEILLEH VTGKGRTFIL AFGGGGGGET QLTDEQCQQL DALLTRRRDG EPIAHLTGVR EFFSLPLFVS PATLIPRPDT ECLVEQALAR LPEQPCR |
| **4xA** | MEFQHWLREA ISQLQASESP RRDAEIAAEH VTGKGRTFIL AFGETQLTDE QCQQADAALT RRRDGEPIAH LTGVREFFSL PLFVSPATLI PRPDTECLVE QALARLPEQP CR |
| **4xA W6F** | MEFQHFLREA ISQLQASESP RRDAEIAAEH VTGKGRTFIL AFGETQLTDE QCQQADAALT RRRDGEPIAH LTGVREFFSL PLFVSPATLI PRPDTECLVE QALARLPEQP CR |

Table 11 Force profile construct of wt HemK full-length, aa sequence N- to C-terminus; numbers indicate construct truncations in HemK aa. Positions for proline substitutions for the HemK Pro variant are shown in red.

| **HemK** | **SecM** | **CspA** |
| --- | --- | --- |
| MEY**Q**HWL**R**EA ISQLQASESP R**R**^22^ DA^24^ EI^26^ **L**L^28^ EH^30^ VT^32^ **G**K^34^ GR^36^ TF^38^ I**L**^40^ AF^42^ GE^44^ TQ^46^ LT^48^ DE^50^ QC^52^ QQ^54^ LD^56^ AL^58^ LT RR^62^ RD^64^ GE^66^ PI^68^ AH L^71^ TG^73^ VR^75^ E^76^ F^77^ W^78^ S^79^ L^80^ P^81^ L^82^ F^83^ V^84^ S^85^ P^86^ A^87^ T^88^ L^89^ I^90^ P^91^ RP^93^ DT^95^ EC^97^ LV^99^ E Q^101^ | FSTPVWIS QAQGIRAGP | MSGKMTGIVK WFNADKGFGFITP |
